# The Usefulness of the CHA_2_DS_2_-VASc Score to Predict Outcomes in Patients with Infective Endocarditis

**DOI:** 10.3390/jcm13164917

**Published:** 2024-08-20

**Authors:** Edward Itelman, Ram Sharony, Ashraf Hamdan, Alaa Atamna, Hila Shaked, Victor Rubchevsky, Yaron D. Barak, Hanna Bernstine, Yaron Shapira, Mordehay Vaturi, Hadass Ofek Epstein, Ran Kornowski, Katia Orvin

**Affiliations:** 1 Cardiology Division, Rabin Medical Center, Petach Tikva 49100, Israelkatiaorvin@gmail.com (K.O.); 2 Tel Aviv Faculty of Medicine, Tel Aviv University, Tel Aviv 69978, Israel; ramsha59@gmail.com (R.S.);; 3 Cardiothoracic Surgery Department, Rabin Medical Center, Petach Tikva 49100, Israel; 4 Infectious Diseases Unit, Rabin Medical Center, Petach Tikva 49100, Israel; 5 Nuclear Medicine Department, Rabin Medical Center, Petach Tikva 49100, Israel

**Keywords:** endocarditis, CHA_2_DS_2_-VASc, risk prognostication, endocarditis team

## Abstract

**Introduction:** Despite diagnostic and therapeutic advances, infective endocarditis (IE) is still associated with high mortality rates. Currently, there are no good prognostic tools for the risk assessment of patients with IE. The CHA_2_DS_2_-VASc score, used to estimate the risk of ischemic stroke in patients with non-valvular atrial fibrillation (AF), has been shown to be a powerful predictor of stroke and death in patients without known AF associated with other cardiovascular conditions. **Objective:** We aimed to evaluate the usefulness of the CHA_2_DS_2_-VASc score as a prognostic tool in a population of patients with IE. **Methods:** The Rabin Medical Center Endocarditis Team (RMCET) registry is a retrospective cohort of all patients evaluated at our center due to acute or sub-acute bacterial endocarditis. The CHA_2_DS_2_-VASc score was extracted for all patients. All-cause mortality was depicted for all patients. **Results:** The cohort included 330 patients with a mean age of 65.2 ± 14.7 years (70% men). During a median follow-up of 24 months [IQR 4.7–48.6], 121 (36.7%) patients died. The median CHA_2_DS_2_-VASc score was 3, and any score above 2 was associated with increased overall mortality (50.8% vs. 19.9%, *p* < 0.001). A multivariate model incorporating important confounders not included in the CHA_2_DS_2_-VASc model showed consistent results with a risk increase of 121% for the higher CHA_2_DS_2_-VASc score groups (HR 2.21 [CI 1.12–4.39], *p* = 0.023). **Conclusions:** IE currently has no good risk stratification models for clinical practice. The CHA_2_DS_2_-VASc score might serve as a simple and available tool to stratify risk among patients with IE.

## 1. Introduction

Despite advances in modern medicine, the in-hospital mortality rate of patients with infective endocarditis (IE) has remained essentially unchanged over the past two decades, ranging from 15% to 30% [1]. This high mortality rate underscores the seriousness of this life-threatening infection and its potential complications, which include heart failure, systemic embolism (including stroke), and death [2]. Managing IE is challenging due to its diverse etiology, unpredictable clinical course, and the complex interplay of host, pathogen, and healthcare-related factors [3]. Thus, developing reliable prognostic tools is crucial for the rapid identification of patients at the highest risk, the timely initiation of therapy and referral to surgery, and potentially improving outcomes. Four main factors affect prognosis at admission: patient characteristics, the presence or absence of cardiac and non-cardiac complications, the infecting microorganism, and echocardiographic findings. Currently, tools for risk estimation are mostly focused on the setting of surgical treatment for IE, though some, such as the Costa Score or the ICE Score, include medically treated patients in their cohorts [4].

The CHA_2_DS_2_-VAS_C_ [congestive heart failure, hypertension, age ≥ 75 years, diabetes, prior stroke, vascular disease, age 65–74 years, sex (female) category] score, which is used to estimate the risk of ischemic stroke in patients with non-valvular atrial fibrillation (AF) [4], is also a powerful predictor of death in patients without known AF [5] in association with other cardiovascular conditions including pulmonary embolism, coronary artery disease, and following transcatheter aortic valve replacement, and even in-hospital mortality of IE patients [5,6,7,8].

However, the utility of the CHA_2_DS_2_-VASc score for the long-term outcomes of patients with IE remains unexplored.

We aimed to evaluate the role of the CHA_2_DS_2_-VASc score as a quick and straightforward prognostication tool in patients diagnosed with IE for mortality.

## 2. Methods

### 2.1. Study Population

This is a retrospective study of all adult patients (>18 years old) treated for the definite diagnosis of IE in the Rabin Medical Center between January 2016 and January 2023. We only excluded patients with incomplete records for analysis. Data were collected from the Rabin Medical Center Endocarditis Team (RMCET) registry, a multidisciplinary approach to treating patients suffering from IE. The Institutional Review Board of the Rabin Medical Centre approved this study based on strictly maintaining participants’ anonymity during database analyses. No individual consent was obtained.

#### 2.1.1. The “Endocarditis Team”

Details of the RMCET were previously reported [9]. In brief, the RMCET was established in January 2016 and consists of general cardiologists, echocardiography specialists, cardiac imaging specialists, nuclear medicine specialists, infectious disease specialists, and cardiac surgeons. Additional consultants (electrophysiology specialists, adult congenital heart disease specialists, nephrologists, and the stroke team) accompanied the team. Any suspected IE case requiring further decision-making, such as establishing a definite diagnosis, requiring further evaluation, or deciding on invasive or conservative management, was referred to the team. The team would then assemble to discuss the cases in depth. The patients were presented during these meetings, clinical data were discussed, and the imaging modalities were revised. The team then decided on further evaluation or the management strategy with the proper recommendations, including post-hospitalization follow-up. The clinical course was evaluated by the team members and reviewed by the ET as needed. All patients were followed up at the cardiology clinics 1–6 months following discharge from the hospital.

#### 2.1.2. The CHA_2_DS_2_-VAS_C_ Score

Based on the CHA_2_DS_2_-VAS_C_ score, patients were given 1 point for congestive heart failure, hypertension, age 65 to 74 years, diabetes mellitus, vascular disease, and female sex, and 2 points for age 75 years or older and previous stroke [9]. Patients were stratified according to their CHA_2_DS_2_-VAS_C_ score into two categories: 0–2 (low score) and 3–9 (intermediate and high score). We assessed the correlation between the CHA_2_DS_2_-VAS_C_ score and clinical outcome, which included stroke, all-cause mortality, and the combined outcome of stroke and/or mortality up to 1 year of follow-up.

### 2.2. Clinical Data and Study Endpoint

The patient’s baseline demographic and clinical data were retrieved from the patient’s computerized records. Clinical data were available for 100% of the complete cohort. Diagnoses were based on computerized hospitalization records (International Classification of Diseases, Ninth Revision [ICD-9] codes), laboratory tests, medications, physiological signals (e.g., ECGs), radiological images (e.g., echocardiograms, angiograms), and procedures’ reports. The primary outcome of the current study was all-cause mortality. Survival data were available for all subjects from the Israeli Population Register up to the end of the follow-up period.

### 2.3. Statistical Analysis

After analyzing the data, continuous variables were expressed as the mean ± standard deviation if normally distributed or the median with interquartile range if skewed. Categorical variables were presented as frequency (%). Continuous data were compared with the Student’s *t*-test, and categorical data were compared using the chi-square or Fisher exact tests. For survival analysis, patients were censored in the case of death; all-cause mortality was available from the national mortality registry. The probability of death according to the study groups was graphically displayed according to the Kaplan–Meier method, with a comparison of cumulative survival across strata by the log-rank test. Univariate Cox proportional hazards regression modeling was used to determine the unadjusted hazard ratio (HR) for all-cause mortality in patients with IE. A multivariate analysis was then performed to evaluate the effect of possible confounders on the primary study endpoint. The multivariate analysis included variables that proved significant in the univariate analysis or that are clinically known to be significant. The following variables were chosen: age, sex, ischemic heart disease, atrial fibrillation, diabetes mellitus, hypertension, acute kidney injury, and embolic stroke. Additionally, the CHA_2_DS_2_VAS_C_ score was assessed as a continuous variable in logistic regression models for 1-year mortality. We did not use imputation or any other method to replace missing values.

All analyses were performed in R software version 4.1 (R Foundation for Statistical Computing). An association was considered statistically significant for a two-sided *p* value of less than 0.05.

## 3. Results

**Figure 1:**
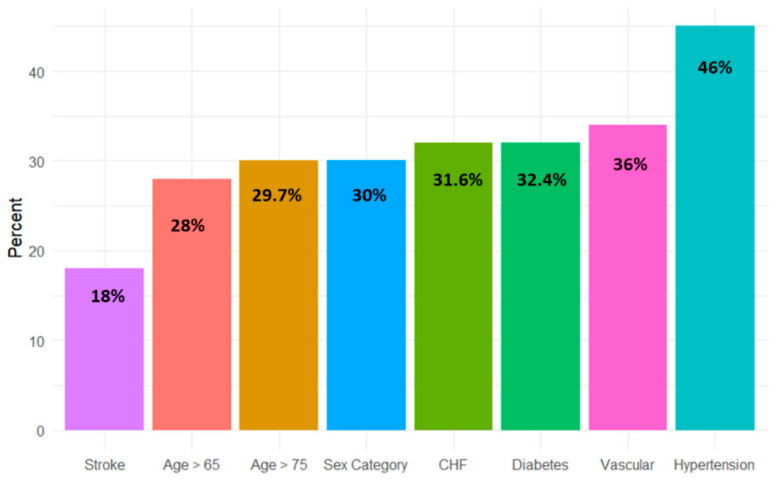
CHA_2_DS_2_-VAS_C_ score components frequency distribution in the study cohort. CHF—congestive heart failure; CVA—cerebrovascular event; DM—diabetes mellitus; HTN—hypertension.

Overall, there were 330 patients with a definite diagnosis of IE during the study period. Their baseline characteristics are presented in Table 1. The mean age was 65.2 ± 14.7 years, and 70% were males. Concerning the CHA_2_DS_2_-VASc parameters, 111 (34%) of patients suffered from ischemic heart disease, 150 (46%) patients suffered from hypertension, 61 (18.4%) patients had a history of stroke, 107 (32.4%) suffered from diabetes, and 104 (31.5%) had a diagnosis of congestive heart failure (Table 1 and Figure 1). A history of AF was present in 55 (16.7%) of patients. Concerning predisposing and risk factors for IE, 137 (46%) of the patients had prosthetic valves, 51 (15.5%) of patients had cardiovascular implantable electronic devices (CIED), 27 (8.2%) had intravascular catheters, and 23 patients (7%) suffered from prior IE (Table 1).

The features and characteristics of IE are presented in Table 2. In addition to standard transthoracic echocardiography (TTE), 278 (84%) patients underwent trans-esophageal echocardiography (TOE), 95 (28.8%) underwent cardiac computer tomography (CT), and 96 (29.1%) underwent FDG PET-CT. Positive IE imaging was observed in 63 (19.1%) of cardiac CT and 35 (10.6%) in FDG PET CT. The most common bacterial pathogens were methicillin-susceptible Staphylococcus aureus (13.9%), enterococcus species (12.1%), coagulase-negative staphylococci (11.2%), and streptococcus species (17.9%) (Table 2).

### 3.1. CHA_2_DS_2_-VAS_C_ Score

**Figure 2:**
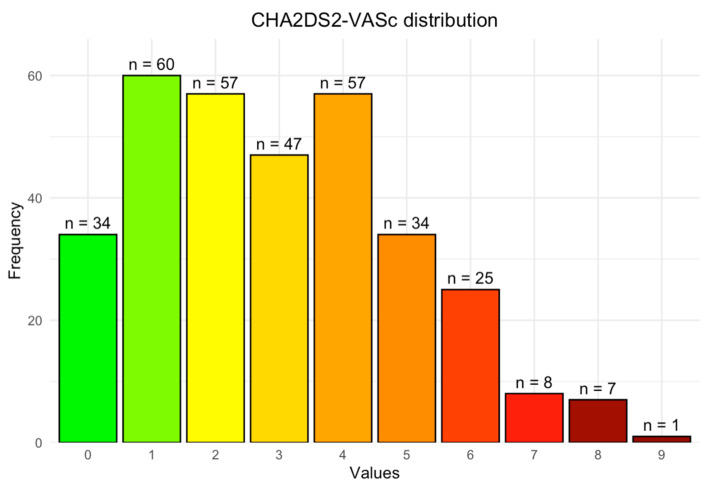
CHA_2_DS_2_-VAS_C_ score frequency distribution in the study cohort.

The median CHA_2_DS_2_-VAS_C_ score was 3 (IQR 1–4). The CHA_2_DS_2_-VAS_C_ score distribution and frequency of the individual components are presented in Figure 1. The distribution of the CHA_2_DS_2_-VAS_C_ score in the study population showed that the most frequent score was 1 (*n* = 60), then 2 and 4 (*n* = 57 for both). The full distribution is displayed in Figure 2. Hypertension and past medical history of vascular disease were the study population’s most common individual score components (Figure 1).

Naturally, patients with CHA_2_DS_2_-VASc scores higher than 2 (Table 1) were older (72.9 ± 10 vs. 56 ± 14, *p* < 0.001) and more likely to be females (40% vs. 18%, *p* < 0.001). Also, patients with higher CHA_2_DS_2_-VASc suffered more frequently from AF (24.6% vs. 7.3%, *p* < 0.001), chronic kidney disease (34.5% vs. 16.1%, *p* = 0.021), and a history of malignancy (15.6% vs. 6.6%, *p* = 0.017). Patients with higher CHA_2_DS_2_-VASc had a higher prevalence of prosthetic valves, mainly percutaneous valves (17.3% vs. 1.3%, *p* < 0.001) and CIED (21.2% vs. 8.6%, *p* = 0.003). They were likelier to undergo TOE examination (92% vs. 75%, *p* < 0.001) but were less frequently referred to cardiac CT (23% vs. 35.8%, *p* = 0.014). They had lower initial hemoglobin results (10.87 ± 2.0 vs. 11.44 ± 2.23, *p* = 0.016) but did not differ in creatinine level, white blood cell count, albumin level, or initial CRP (Table 2).

**Figure 3:**
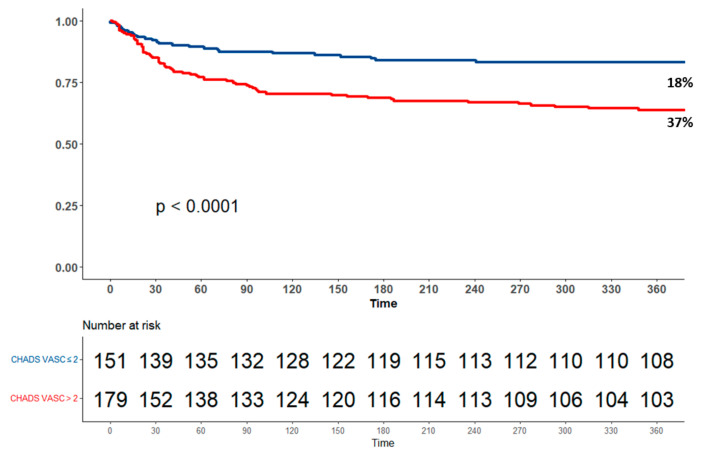
Kaplan–Meier survival curves as stratified for CHA_2_DS_2_-VAS_C_ score according to group category—0–2 (low score), 3–9 score (intermediate to high score).

Patients with higher CHA_2_DS_2_-VASc were less likely to undergo surgical intervention (21.8% vs. 40%, *p* < 0.001) and thus more likely to be managed conservatively (78% vs. 60%, *p* < 0.001). The incidence of embolic stroke was not higher in patients with higher CHA_2_DS_2_-VASc (11.2% vs. 17.2%, *p* = 0.156). During a median follow-up of 24 months [IQR 4.7–48.6], 121 (37%) patients died (Table 3). In-hospital and long-term mortality were significantly higher in the high CHA_2_DS_2_-VASc group (19.6% vs. 8.6%, *p* = 0.008, and 50.8% vs. 19.9%, *p* < 0.001, respectively). Kaplan–Meier survival analysis showed that the cumulative probability of death among patients with and without higher CHA_2_DS_2_-VASc scores was significantly higher in patients with CHA_2_DS_2_-VASc > 2, with one-year survival rates of 63.79% in the CHA_2_DS_2_-VASc group and 83.2% in the low CHA_2_DS_2_-VASc group (p log-rank < 0.001 for the overall difference during follow-up) (Figure 3). A univariate Cox regression model showed that compared with lower CHA_2_DS_2_-VASc scores, patients with CHADS-VASC scores > 2 had almost 3-fold higher mortality rates during follow-up (HR 2.93, CI 1.94–4.43, *p* < 0.001) (Table 4).

When tested as a continuous variable in a logistic model to predict long-term outcomes, each 1-point increase in the CHA_2_DS_2_-VAS_C_ score was associated with a 23% relative increase in the risk of mortality (CI 1.13–1.35, *p* < 0.001).

### 3.2. Multivariate Analysis

Other properties considered major contributing factors to mortality were integrated into a multivariate analysis to eliminate all significant confounders. This analysis is displayed in Table 5. According to the multivariate analysis, statistically significant predictors of mortality remained CHA_2_DS_2_-VASc score > 2 (HR 2.21, CI 1.12–4.39, *p* = 0.023) and acute kidney injury (HR 2.18, CI 1.44–3.30, *p* < 0.001). The presence or absence of baseline AF or complication of embolic stroke did not modify the predictive value of the CHA_2_DS_2_-VAS_C_ score on outcomes (Table 5).

## 4. Discussion

Our study aimed to evaluate whether the CHA_2_DS_2_-VAS_C_ score could be a practical tool for predicting mortality in IE (regardless of AF background). Our study’s main findings were as follows: (1) Patients with higher scores had more co-morbidities, including chronic kidney disease, percutaneous prosthetic valve, and CIED. (2) Patients with higher CHA_2_DS_2_-VASc were less likely to undergo surgical valve replacement and more likely to be managed conservatively. (3) Compared to the low score groups, patients with a CHA_2_DS_2_-VAS_C_ score > 2 were associated with a twice as high incidence of in-hospital and one-year mortality and a 3-fold increase overall mortality risk. (4) Each 1-point rise in the CHA_2_DS_2_-VAS_C_ score was associated with a 23% relative increase in mortality.

Contemporary long-term survival rates in patients who completed IE treatment are estimated to be 85–90% and 70–80% at 1 and 5 years, respectively [9].

Four main factors affect prognosis in IE: patient characteristics, the presence or absence of cardiac and non-cardiac complications, the infecting microorganism, and echocardiographic findings.

The main predictors for long-term mortality, considering patients’ characteristics and co-morbidities, include older age, diabetes mellitus, hemodialysis, a high Charlson comorbidity index, and heart failure. The 2023 European Society of Cardiology Guidelines for the management of IE emphasize the importance of a multidisciplinary approach in the form of an “endocarditis team”, recommending prognostic assessment to identify patients at higher risk of changing the course of their disease. However, according to contemporary literature and clinical practice, no prognostic tool is available that encompasses the main predictors that could be collected early following admission.

We chose to investigate a pre-existing score, the CHA_2_DS_2_-VAS_C_ score, which is well established and routinely used in daily practice among physicians and cardiologists. Initially used for risk assessment in patients with atrial fibrillation, the CHA_2_DS_2_-VAS_C_ score has been investigated and proven useful in mortality risk assessment in other cardiovascular conditions [6,7,8]. We have previously shown a good correlation between the CHA_2_DS_2_-VAS_C_ score and long-term mortality risk in patients undergoing percutaneous coronary intervention [10] and transcatheter aortic valve implantation [7], and a higher score was associated with increased in-hospital mortality of endocarditis [8]. Following these findings, we explored the potential of the CHA_2_DS_2_-VAS_C_ score as a predictor of mortality in the IE population.

Several facts underlie the rationale for this choice. First, the CHA_2_DS_2_-VAS_C_ score has several components that overlap with predictors for long-term mortality (older age, diabetes, congestive heart failure, and embolic stroke) [9]. Second, the CHA_2_DS_2_-VAS_C_ score is a well-known, simple, and practical risk score widely applied at the bedside and does not require computerized calculations.

Previous studies have evaluated clinical characteristics associated with mortality in IE, focusing on in-hospital mortality primarily attributed directly to IE complications [11]. Longer-term studies have demonstrated higher mortality rates in IE patients beyond the index hospitalization, predominantly due to non-cardiovascular reasons [12,13]. As mentioned, multiple factors influencing in-hospital and long-term mortality, combining clinical, microbiological, and imaging characteristics, make risk assessment in the IE complex.

Published risk scores have been limited to small studies in tertiary centers, excluding patients with CIED or prosthetic valves or small surgical cohorts and short-term follow-ups [14].

Park L et al. developed a simplified independent and weighted prognostic score for IE for clinical use from the international collaboration of the IE registry [15]. The prognostic factors for mortality were categorized into four variables: host factors, IE characteristics, complications, and treatment with or without surgery [16]. Although they developed a simplified risk model, it has not yet been adopted for daily practice, probably due to its complex scoring and calculation methods. In this study, patient age and complications of IE, particularly heart failure symptoms and embolic stroke [17], were found to be the strongest predictors of mortality. In another study [18], diabetes mellitus, and especially insulin-dependent diabetes mellitus, was shown to be strongly associated with high mortality rates in IE. Combining these well-established individual risk factors in the CHA_2_DS_2_-VAS_C_ score may be sufficient for a rapid risk stratification estimate, as shown by Abe et al. [8] in the short-term hospital stay and by us in the long-term in this study. The ideal risk score should have simple, measurable parameters comparable across centers to ensure widespread use.

The strength of our study is the contemporary cohort of IE patients who were managed by an “Endocarditis Team” of experts in a tertiary cardiac center, comprising mixed high-risk patients with a high prevalence of prosthetic valves, CIED, and co-morbidities and including patients managed conservatively as well as surgically with a long-term follow-up of up to 5 years.

## 5. Limitations

Our study has several limitations. First, this study is based on a relatively small single-center cohort. However, registries that represent daily practice may be more appropriate for risk score adjustment. Second, we did not perform a validation analysis or compare the CHA_2_DS_2_-VAS_C_ score against other available risk stratification scores. We rely on the fact that age, diabetes mellitus, heart failure, and stroke are established risk factors previously shown to predict worse outcomes in IE.

## 6. Conclusions

We have shown that the CHA_2_DS_2_-VAS_C_ score is a simple and effective tool to predict mortality in IE patients. This simple tool could be widely applicable to predict patient outcomes and guide intervention. This study reinforces previous studies that demonstrated the efficiency and simplicity of this risk stratification tool in multiple cardiovascular scenarios. Further studies to prospectively validate the use of the CHA_2_DS_2_-VAS_C_ score in endocarditis patients are required.

## Figures and Tables

**Table 1:** Baseline characteristics. Values are presented as number (%) or median [IQR].

	Overall	CHADS-VASC 0–2	CHADS-VASC > 2	*p* Value
**N**	330	151	179	
**Age (years)**	65.2 ± 14.7	56 ± 14.2	72.9 ± 10	<0.001
**Gender—male**	232 (70)	124 (82)	108 (60)	<0.001
**Diabetes mellitus**	107 (32)	13 (8.6)	94 (52.5)	<0.001
**Hypertension**	150 (46)	33 (21.9)	117 (65)	<0.001
**Coronary artery disease**	111 (34)	15 (9.9)	96 (53.6)	<0.001
**Heart failure**	104 (32)	28 (18.5)	76 (42.5)	<0.001
**Prior stroke**	61 (21.3)	4 (3.4)	57 (33.5)	<0.001
**Atrial fibrillation**	55 (16.7)	11 (7.3)	44 (24.6)	<0.001
**Chronic kidney disease**	40 (26.8)	10 (16.1)	30 (34.5)	0.021
**Malignancy (active or past)**	38 (11.5)	10 (6.6)	28 (15.6)	0.017
**Prosthetic valve**	137 (42)	50 (33.1)	87 (48.6)	0.006
Mechanical	55 (16.7)	23 (15.2)	32 (17.9)	0.621
Biological	21 (6.4)	14 (9.3)	7 (3.9)	0.078
Percutaneous valve (TAVI, Melody)	33 (10)	2 (1.3)	31 (17.3)	<0.001
**Prior endocarditis**	23 (7)	7 (4.6)	16 (8.9)	0.189
**Cardiovascular implantable electronic device** (Pacemaker/CRT/ICD)	51 (15.5)	13 (8.6)	38 (21.2)	0.003
**Vascular catheters** (Piccline/portacath/permacath)	27 (8.2)	7 (4.6)	20 (11.2)	0.05

CRT—cardiac resynchronization therapy; ICD—implantable cardioverter defibrillator; TAVI—transcatheter aortic valve implantation.

**Table 2:** Infective endocarditis features. Values are presented as number (%) or median [IQR].

	Overall	CHADS-VASC 0–2	CHADS-VASC > 2	*p* Value
**N**	330	151	179	
**Fever (max, °C)**	37.9 ± 0.9	37.8 ± 0.9	38 ± 0.9	0.213
**CRP (highest)**	16.481 ± 0.75	16.62 ± 10.94	16.37 ± 10.62	0.836
**Hemoglobin (on presentation, g/dL)**	11.13 ± 2.12	11.44 ± 2.23	10.87 ± 1.99	0.016
**Creatinine (on presentation, mg/dL)**	1.62 ± 1.53	1.46 ± 1.52	1.75 ± 1.53	0.082
**Platelets (on presentation)**	210 ± 96	213 ± 102	207 ± 91	0.541
**Albumin (on presentation, g/dL)**	3.57 ± 0.62	3.64 ± 0.66	3.51 ± 0.58	0.058
**WBC (on presentation, k/µL)**	10.91 ± 5.8	10.68 ± 5.85	11.1 ± 5.76	0.513
**CRP (on presentation)**	10.79 ± 8.97	11.05 ± 9.41	10.56 ± 8.61	0.625
**Cardiac CT**	95 (28.8)	54 (35.8)	41 (22.9)	0.014
**FDG—PET—CT**	96 (29.1)	43 (28.5)	53 (29.6)	0.917
**Trans-esophageal echocardiography**	278 (84)	113 (75)	165 (92)	<0.001
**Ejection fraction—median (IQR)**	60 (45–60)	60 (55–60)	60 (40–60)	0.009
**Echo abscess**	49 (14.8%)	28 (18.5%)	21 (11.7%)	0.521
**Echo vegetation**	191 (57.9%)	95 (62.9%)	95 (53.1%)	0.071
**Endocarditis on prosthetic valve**				
**Positive IE imaging on FDG PET CT**	35 (10.6)	20 (13.2)	15 (8.4)	0.211
**Positive IE imaging cardiac CT**	151 (46)	74 (49)	77 (43)	0.328
**Microbiology**				0.001
**Enterococcus**	40 (12.1)	11 (7.3)	29 (16.2)	
**Fungi**	3 (0.9)	1 (0.7)	2 (1.1)	
**Gram-negative bacteria**	16 (4.8)	5 (3)	11 (6.7)	
**CoNS**	37 (11.2)	15 (9.9)	22 (12.3)	
**HACEK group**	13 (3.9)	8 (5.3)	5 (2.8)	
**MRSA**	14 (4.2)	3 (1.8)	11 (6.7)	
**MSSA**	46 (13.9)	19 (12.6)	27 (15.1)	
**Other strep species**	59 (17.9)	26 (17.2)	33 (18.4)	
**Others**	64 (19.4)	45 (29.8)	19 (10.6)	
**Q FEVER**	11 (3.3)	5 (3.3)	6 (3.6)	
**Strep viridans**	27 (8.2)	14 (9.3)	13 (7.3)	

CT—computer tomography; MSSA—methicillin-sensitive staph aureus; MRSA—methicillin-resistant staph aureus; HACEK—Haemophilus species, *Aggregatibacter actinomycetemcomitans*, *Cardiobacterium hominis*, *Eikenella corrodens*, *Kingella kingae*.

**Table 3:** IE management and outcome. Values are presented as number (%) or median [IQR].

	Overall	CHADS-VASC ≤ 2	CHADS-VASC > 2	*p* Value
**N**	330	151	179	
**Surgical valve replacement/repair**	102 (31)	63 (41.7)	39 (21.8)	<0.001
**Conservative management**	228 (69)	88 (58.3)	140 (78)	<0.001
**In-hospital brain emboli and stroke**	46 (13.9)	26 (17.2)	20 (11.2)	0.156
**In-hospital acute kidney injury**	93 (33.9)	29 (25.7)	64 (39.8)	0.022
**In-hospital mortality**	48 (14.5)	13 (8.6)	35 (19.6)	0.008
**1-year mortality**	88 (26.7)	24 (15.9)	64 (35.8)	<0.001
**Hospital stay (days)**	19.57 (16.08)	18.57 (15.66)	20.62 (16.51)	0.25
**Overall mortality (study period)**	121 (36.7)	30 (19.9)	91 (50.8)	<0.001

**Table 4:** Long-term outcomes predicted for each 1 increase in incremental score.

	HR (CI)	*p* Value
**CHA_2_D_S_-VASC 1**	1.44 [0.51, 4.05]	0.485
**CHA_2_D_S_-VASC 2**	1.61 [0.57, 4.58]	0.37
**CHA_2_D_S_-VASC 3**	3.06 [1.13, 8.24]	0.027
**CHA_2_D_S_-VASC 4**	3.90 [1.51, 10.09]	0.005
**CHA_2_D_S_-VASC 5**	5.87 [2.21, 15.58]	<0.001
**CHA_2_D_S_-VASC 6**	4.94 [1.78, 13.73]	0.002
**CHA_2_D_S_-VASC 7–9**	3.61 [1.18, 11.03]	0.024

**Table 5:** Multivariate Cox model.

	HR (CI)	*p* Value
**CHA_2_D_S_-VASC > 2**	2.21 [1.12, 4.39]	0.023
**Acute kidney injury**	2.18 [1.44, 3.30]	<0.001
**Age**	1.02 [1.00, 1.04]	0.088
**Male**	1.32 [0.85, 2.04]	0.215
**Coronary artery disease**	1.08 [0.68, 1.74]	0.737
**Atrial fibrillation**	0.83 [0.54, 1.28]	0.392
**Diabetes mellitus**	1.33 [0.87, 2.02]	0.189
**Hypertension**	1.22 [0.79, 1.88]	0.368
**Embolic stroke**	1.16 [0.66, 2.04]	0.613

## Data Availability

Data is unavailable due to privacy and ethical restrictions.
